# Raman, Dilatometric, and Dielectric Insights into Pr^3+^-Doped Pb–Sb Silicate Glasses toward Ion-Conducting Glass Electrolytes

**DOI:** 10.1021/acs.jpcb.5c05304

**Published:** 2025-09-22

**Authors:** Yeti Dana Rao, Vandana Ravi Kumar, Luka Pavić, Arijeta Bafti, José A. Jiménez, Ayyagari Venkata Sekhar, Paulina Kapuśniak, Piotr Brągiel, Michal Piasecki, Nalluri Veeraiah

**Affiliations:** † Department of Physics, 28629 Acharya Nagarjuna University, Nagarjuna Nagar, Guntur 522 510, India; ‡ Rud̵er Boskovic Institute, Zagreb HR-10000, Croatia; § Faculty of Chemical Engineering and Technology, University of Zagreb, 10000 Zagreb, Croatia; ∥ Center for Advanced Materials Science, Department of Biochemistry, Chemistry & Physics, 7604 Georgia Southern University, Statesboro, Georgia 30460, United States; ⊥ Faculty of Science and Technology, Jan Dlugosz University, 13/15, Armii Krajowej Aleja, PL-42-200 Częstochowa, Poland

## Abstract

This work reports new physical insights concerning the effect of red lead (Pb_3_O_4_) addition (10–35 mol %) on the structural, dilatometric, dielectric, and conductivity properties of Sb_2_O_3_–SiO_2_:Pr_2_O_3_ glasses for potential solid-state electrolyte applications. The melt-quenched glasses were scrutinized via Raman spectroscopy including a temperature-dependent evaluation revealing progressive polymerization of the glass network up to 30 mol % Pb_3_O_4_, followed by depolymerization at 35 mol %. Harmonizing with the structural evolution, thermal analysis by dilatometry showed that the thermal expansion coefficients/softening temperatures first decreased/increased from 10 to 30 mol % Pb_3_O_4_ and then increased/decreased for 35 mol % Pb_3_O_4_. The dielectric properties and ac conductivity were measured over 0.02–1 MHz and 20–240 °C. An increase in Pb_3_O_4_ from 10 to 30 mol % led to reduced dielectric constant and conductivity, which is attributed to a more compact and polymerized structure that limits ion mobility. Here, conduction is primarily polaronic, supported by mixed-valence Pb^2+^/Pb^4+^ and Sb^3+^/Sb^5+^ ions. At 35 mol % Pb_3_O_4_, network depolymerization introduced nonbridging oxygens and structural disorder, enhancing the free volume and ion migration pathways. Consequently, ionic conduction, particularly of Pb^2+^, becomes dominant, significantly boosting the conductivity. Although Pb^2+^ ions are relatively immobile compared to Li^+^ or Na^+^, the insights gained offer a foundational understanding and guide the development of similar glass systems doped with lighter and more mobile alkali ions for practical battery applications.

## Graphical abstract

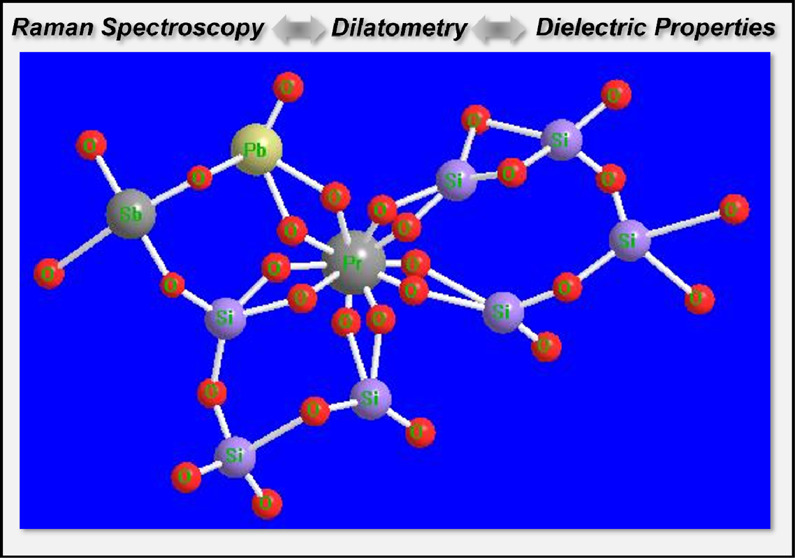

## Introduction

The dielectric properties and ac conductivity of glass materials over long ranges of frequencies and temperatures offer valuable insights into their conduction mechanisms and structural characteristics.^1–5^ These properties are critical in determining the suitability of such materials for various applications, such as electrolytes and electrodes in solid-state batteries, capacitors, insulators, and electronic components, as well as in sensors for measuring physical and chemical parameters, such as temperature, humidity, and gas concentration. Glasses doped with different rare earth (RE) ions (such as Pr, Nd, Er, Yb, etc.) display distinct dielectric behavior due to their unique electronic structures. The concentration of these ions significantly affects the dielectric performance of the materials.^6–11^

Some key applications relating to dielectric studies on glass materials doped with RE ions include: as insulators in capacitors, transformers, and circuit boards because of their high dielectric strength and low loss tangent and thin glass sheets as substrates for integrated circuits and microelectronics.^6^ Low-loss glasses are useful in radomes for protecting antennas and preserving signal integrity. Glass capacitors with RE ions are used for high-frequency and high-voltage applications, offering stability and high breakdown voltage, in energy storage systems such as supercapacitors and batteries, as well as glass fibers for data transmission with low loss over long distances.^12^ Overall, the ability to modify the composition and structure of glass allows researchers to tailor its dielectric properties for specific needs, making it a versatile material in many cutting-edge technologies. Studies along these lines have been carried out on a variety of glass materials doped with different RE ions.^6–12^

As a nontraditional system, antimony silicate glasses containing Pr^3+^ ions are expected to exhibit interesting changes in dielectric properties when admixed with red lead (Pb_3_O_4_). For instance, increasing the lead oxide content generally raises the dielectric constant (since its density increases), while a higher silica content tends to lower it. These properties are also frequency-dependent: at lower frequencies, the dielectric constant is usually higher, and at higher frequencies, in general, the dielectric constant decreases.^13–16,45^ Temperature is another factor that affects the dielectric properties. As the temperature rises, dielectric loss and conductivity tend to increase. The dielectric constant of these glasses varies widely depending on factors, such as the dopant RE ion concentration, matrix composition, and external conditions. These glasses also exhibit dielectric relaxation, where the dielectric constant and loss change with the frequency. The relaxation time is influenced by the type and concentration of RE ions and the glass composition. Such studies are of interest for numerous applications as mentioned above.^13–16,45^ Understanding the insulating behavior and structure of glass materials relies heavily on in-depth studies of their dielectric properties and ac conductivity across wide regions of frequency and temperature. Indeed, several researchers have successfully investigated these aspects in various glasses and glass-ceramics, which provided useful insights.^13–16,45^

Overall, the dielectric properties of RE-doped lead antimony silicate glasses are complex and depend on various factors as mentioned above. A thorough understanding of these properties is essential for the design and optimization of devices that utilize these materials. In this study, we have chosen Pr^3+^ as dopant in the antimony silicate glass system. Pr^3+^-doped glasses are widely recognized for their near-infrared (NIR) laser emission, which finds extensive applications in telecommunications.^17–21^ However, their dielectric properties have been less explored, with only a few studies reported,^22–24^ indicating ample scope for further research in this field. Further, we choose to add a nontraditional lead oxide, viz., red lead (Pb_3_O_4_) as an additional component in antimony silicate glasses containing Pr^3+^ ions. The incorporation of red lead increases the density of these glasses, which is expected to significantly affect their dielectric properties. Furthermore, the inclusion of Pb_3_O_4_ broadens the potential applications of these glasses, such as radiation shielding, optically operated devices, and electrolytes in solid-state batteries. Red lead is a remarkable heavy metal oxide that disintegrates into PbO via α-PbO_2_ and β-PbO_2_ polymorphs at approximately 600 °C.^25,26^

In the α-PbO_2_ structure, the lead atom is surrounded by eight oxygen atoms arranged in an octahedral geometry. These octahedra are linked through the sharing of the corners and edges. In contrast, the β-PbO_2_ polymorph exhibits a different arrangement, where the octahedra share opposite edges and corners. In β-PbO_2_, the corner-sharing octahedra are tilted, resulting in two distinct Pb–O bond lengths: shorter bonds (∼2.17 Å) and slightly longer bonds (∼2.18 Å). Additionally, β-PbO_2_ is a more stable polymorph that is resistant to corrosion, even in acidic environments.^27,28^ This contributes to the formation of the network with PbO_4_ groups and establishes links with SiO_4_ and SbO_3_ units. Both polymorphs of PbO_2_ get reduced to PbO upon electron capture, and the resulting Pb^2+^ ions that act as modifiers, and introduce various imperfections into the glass structure by breaking interconnecting bonds of various structural units in the glass network.^27,28^ As a result, both Pb^2+^ and Pb^4+^ ions are expected to have significant impact on the insulating properties of antimony silicate glasses. Recently, we reported a detailed investigation of the NIR emission of Pr^3+^ ions in this glass system.^29^ The objective of this study is then to shed light into the structure–property relationship by carrying out detailed studies on dielectric properties that include dielectric constant, loss, electric moduli, impedance spectra, and ac conductivity of Sb_2_O_3_–SiO_2_:Pr_2_O_3_ glasses as a function of Pb_3_O_4_ concentration (10–35 mol %) and to analyze the data in connection with structural variations in the glass matrix using Raman spectra and also thermal expansion measurements.

## Materials and Methods

### Glass Fabrication

The following chemical compositions of the glasses with gradual decrement of Pb_3_O_4_ from 35 to 10 mol % were chosen for this investigation. The details of the composition are as follows: (40 – *x*)­Pb_3_O_4_–49Sb_2_O_3_–(10 + *x*)­SiO_2_: 1Pr_2_O_3_ with *x* = 5 (Pb_35_), 10 (Pb_30_), 20 (Pb_20_), and 30 (Pb_10_). Glass materials were synthesized by using the melt-quenching method. High-purity Pb_3_O_4_, Sb_2_O_3_, SiO_2_, and Pr_6_O_11_ (Sigma-Aldrich) in appropriate proportions were accurately weighed, mixed, and melted in platinum crucibles at 1400 °C for 30 min. The molten mixture was rapidly cooled in brass molds and annealed at 350 °C to minimize internal defects such as voids, cracks etc. The amorphous nature of the samples was confirmed by X-ray diffraction; the details of these and other preliminary characterizations by techniques such as optical absorption, IR spectroscopy, and X-ray photoelectron spectroscopy (XPS) can be found in ref 29.

### Measurements

For identifying structural variations in the glass network due to the variation in the content of Pb_3_O_4_, Raman spectra of these glasses were recorded. The spectra of polished glass slabs were obtained at room temperature using a Thermo Scientific DXR Raman microscope (10× objective; 532 nm laser operating at 10 mW). A Renishaw inVia Raman System (integrated with a confocal optical microscope (30% transmission, 250 mm focal length)) was used for recording spectra at higher temperatures within 100–400 °C. The Renishaw inVia Raman spectrometer is a high-performance confocal spectrometer augmented with a Linkam temperature stage, operating between −196 and 1500 °C. It combines a research-grade microscope with an advanced spectrometer, optimized for high-quality spectral data even from small samples, supporting both point analysis and chemical imaging. It consists of two low-noise, high-sensitivity CCD detectors (viz., EMCCD and InGaAs). It contains three excitation sources, viz., 355, 532, and 830 nm, and it is possible to record the spectra from deep UV to near-IR regions with the minimal fluorescence and resonance enhancement. The system supports micrometer-resolution, 3D mapping, and advanced imaging modes such as Stream Line, Stream HR, and True Raman Imaging. Live Track enables real-time autofocus for curved or uneven surfaces without prior topography scans, ensuring accurate 3D and spectral data. In this study, the 532 nm laser with 10 mW power was used as the excitation source, and the spectra were recorded with 50× MPlan objective lens and 2400 L/mm grating. The spectra were captured with a 10 s acquisition time at four distinct elevated temperatures (100, 200, 300, and 400 °C) and processed with Origin Pro for baseline correction and normalization.

For identifying the proportions of Pb^2+^ and Pb^4+^ ions in the glass composition, X-ray photoelectron spectra (XPS) were recorded using a PHI 5000 Versa Probe ULVAC instrument equipped with a monochromatic Al Kα source operating at an energy of 1486.6 eV with respect to the C 1s peak at 284.6 eV, as discussed in ref 29.

The thermal analysis by dilatometry was carried out on the glasses cast as rods using an Orton dilatometer (Model 1410B) operating in an ambient atmosphere at a heating rate of 3 °C/min. The thermal expansion data obtained was employed to determine the coefficient of thermal expansion (CTE) within 50–350 °C and the dilatometric or softening temperature (*T*_s_) through the instrument’s software.

For dielectric and ac conductivity measurements, both surfaces of the glass samples were coated with thin gold electrodes of approximately 6 mm in diameter using a sputter coater SC7620 by Quorum Technologies. Measurements were carried out using a Novocontrol Alpha-AN dielectric spectrometer across a frequency range of 0.04 Hz–1.0 MHz and a temperature range of 20–240 °C, with 5 °C intervals (±0.2 °C accuracy). The resulting impedance spectra were evaluated by using equivalent circuit (EC) modeling.

## Results and Discussion

A brief summary of the results from the XPS, IR, and optical absorption studies reported in ref 29 is presented herein first so as to facilitate the analysis of the results of dielectric measurements. The analysis via XPS in the various binding energy regions within 136–144 eV (4f region of lead ions) and 528–544 eV (3d region of antimony ions) indicated a gradual increase in the concentration of Pb^4+^ ions (participate and interconnect with SiO_4_ units in the glass network) at the expense of Pb^2+^ ions (act as modifiers). Similarly, the concentration of Sb^5+^ ions that are predicted to participate in the glass network with Sb^V^O_4_ units^30^ has been observed to increase gradually at the expense of Sb^3+^ ions, which act as modifiers. From the XPS studies, it was concluded that there is an increasing degree of rigidity of the glass network with an increase in the Pb_3_O_4_ concentration in the glass matrix.^29^ The IR spectral analysis also indicated a similar inference. We have observed a gradual increase in the intensity of various conventional symmetrical vibrational bands of SiO_4_ and Sb^V^O_4_ units, whereas the asymmetrical vibrational bands of these units exhibited a decrease with an increase of Pb_3_O_4_ beyond 10 mol %.^29^

### Raman Spectroscopy

Seeking additional insights and to investigate further the structural properties, detailed measurements were conducted herein by Raman spectroscopy. As a starting point for comparing the various glasses, Figure 1 presents Raman spectra obtained at room temperature in the wavenumber region 300–1400 cm^–1^. The spectra exhibit at low frequencies a prominent band within 330–560 cm^–1^ (referred to as the D1 band), attributed to the bending vibrations of Si–O bonds in four-membered siloxane rings.^13,31^ The intensity of this band was observed to decrease with increasing Pb_3_O_4_ concentration from 10 to 35 mol %. In this region, the ν_4_ (doubly degenerate bending) vibrations of Sb–O bonds are also possible.^32^ Another band is located in the region within 615–650 cm^–1^ (D2 band), associated with bending vibrations of Si–O bonds in three-membered siloxane rings.^13,31^ The intensity of this band appears to increase proportionally with higher Pb_3_O_4_ levels from Pb_10_ to Pb_35_, while it displays a shift toward higher frequencies. It is likely that the ν_2_ (doubly degenerate bending) vibrations of Sb–O bonds contribute to this region, along with potential overlap from the vibrations of Pb–O bonds from Pb^IV^O_4_ structural units.^32,33^ A strong band that diminishes with Pb_3_O_4_ content from 10 to 30 mol % was also detected around 980 cm^–1^. This band represents the symmetric stretching of Si–O^–^ (nonbridging oxygen sites) bonds.^31^ When Pb_3_O_4_ is raised from 30 to 35 mol %, this band appears to dominate. This region may also accommodate the symmetric stretching (ν_1_) vibrational band of Sb–O bonds. Additionally, a weak feature around 1101 cm^–1^ is visualized, and this is identified as arising from asymmetric stretching vibrations of Si–O–Si linkages.^34^

**Figure 1:**
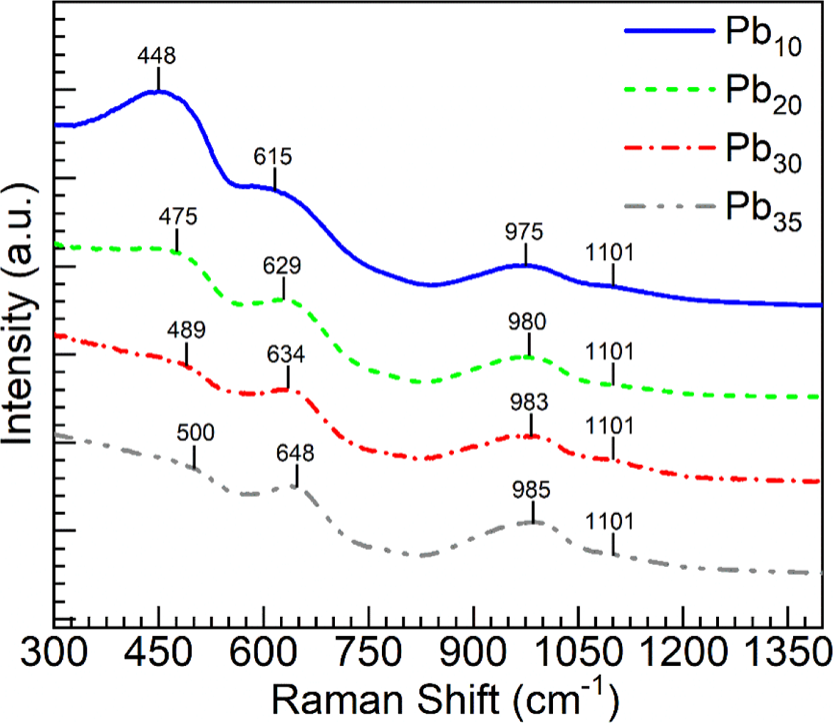
Comparison of Raman spectra of Pb_3_O_4_–Sb_2_O_3_–SiO_2_:Pr_2_O_3_ glass ceramics mixed with different concentrations of Pb_3_O_4_ as recorded at room temperature with the Thermo Scientific DXR Raman microscope operating at 532 nm with a power of 10 mW.

Elevated-temperature Raman spectroscopy of glass materials provides insights into the thermal stability, phase behavior, bond dynamics, and structural transitions, making it a powerful tool to study the integrity and evolution of the glass network under thermal stress.^35–37^ It is with this view that we have performed Raman spectra of these glasses at elevated temperatures. Figure 2a–d represent the Raman spectra recorded for different glasses at different temperatures (100, 200, 300, and 400 °C) with the Renishaw inVia Raman System. A summary of the Raman band positions observed at various temperatures is provided in Table 1. Herein, we have noticed a prominent band at about 151 cm^–1^, which is likely associated with Pb–O–Pb bending or lattice modes involving Pb–O clusters.^38–42^ The intensity of this band is gradually decreased with an increase of the Pb_3_O_4_ content from 10 to 30 mol %. In this concentration range, there is a possibility for fewer Pb–O–Pb clusters in favor of interconnected Pb–O units or [PbO_4_] pyramidal units that reinforce Pb–O–Pb bending modes and reduce the intensity of the 151 cm^–1^ band. Therefore, the decrease in the band intensity corresponds to progressive polymerization and reduced collective Pb–O–Pb vibrations. However, an abnormal hike is observed when the content of Pb_3_O_4_ is raised from 30 to 35 mol %. This observation suggests a shift from a network former/modifier balance to Pb–O cluster dominance, or even incipient phase separation, which is well supported in the glass science literature.^38–42^

**Figure 2:**
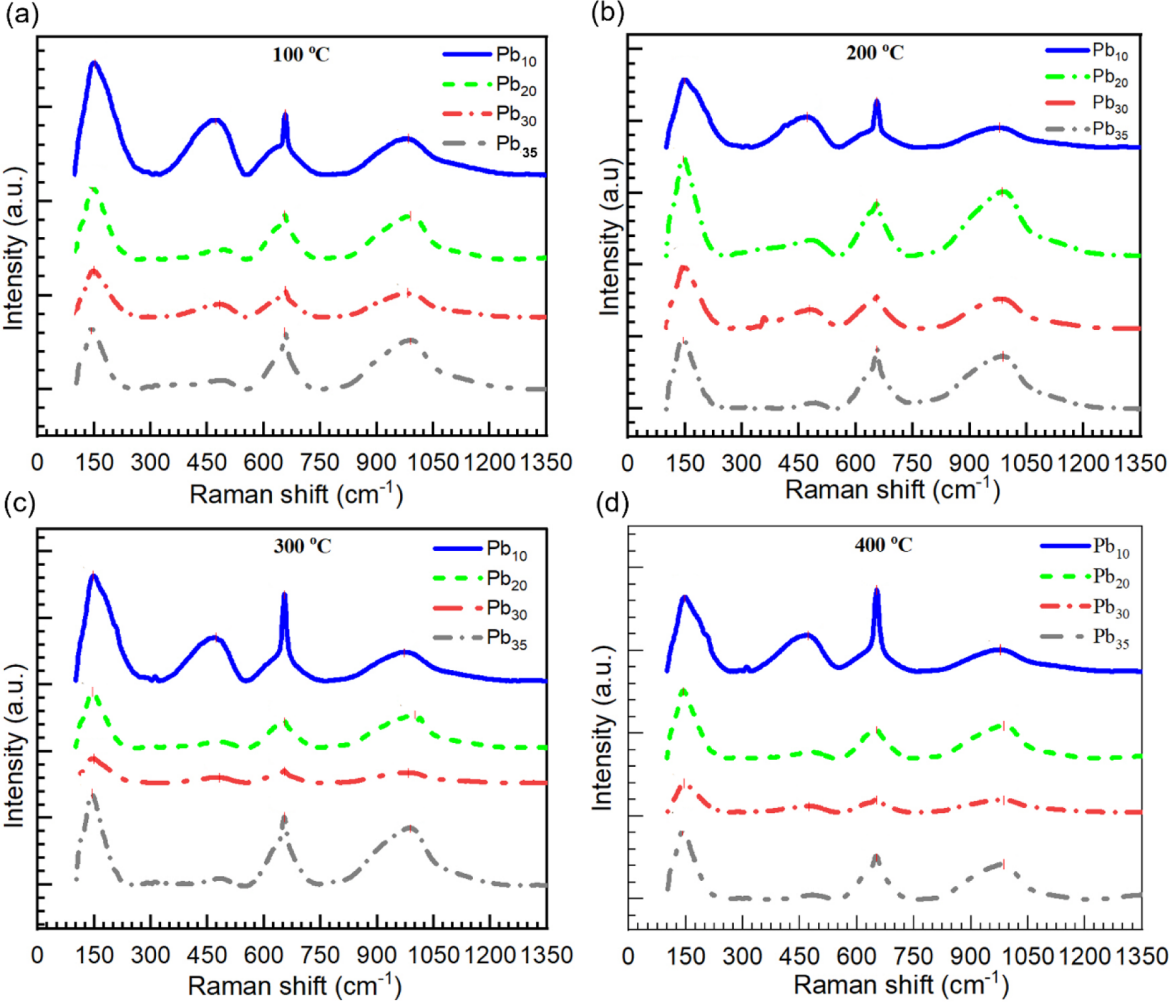
Raman spectra of Pb_3_O_4_–Sb_2_O_3_–SiO_2_:Pr_2_O_3_ glasses mixed with various concentrations of Pb_3_O_4_ recorded at (a) 100 °C, (b) 200 °C, (c) 300 °C, and (d) 400 °C. Band positions are summarized in Table 1.

**Table 1:** Summary of the Data on Raman Spectra of Pr^3+^-Doped Pb_3_O_4_–Sb_2_O_3_–SiO_2_ Glasses^a^

glass	Pb_10_				Pb_20_				Pb_30_				Pb_35_			
vibrational bands / positions at different temperatures	100 °C	200 °C	300 °C	400 °C	100 °C	200 °C	300 °C	400 °C	100 °C	200 °C	300 °C	400 °C	100 °C	200 °C	300 °C	400 °C
Pb–O–Pb bending	152	145	149	142	149	145	147	145	147	145	144	143	147	143	145	142
Si–O bond (4-membered siloxane rings)/ν_4_ Sb–O	472	474	483	485	473	478	477	478	472	484	482	484	472	475	474	476
Si–O bond (3-membered siloxane rings)/ν_2_ Sb–O/Pb–O vibs Pb^IV^O_4_ units	657	656	657	656	656	655	655	655	653	653	653	653	652	651	651	651
Si–O^–^ vibs/ν_1_ Sb–O asymmetric stretchings of Si–O–Si linkage	985	989	983	990	980	987	986	988	971	1000	982	988	977	987	986	986

a The band positions are given in cm^–1^.

With the increase in the temperature of the measurements, changes are observed in the Raman spectra (Figure 2) as follows. When the temperature is raised to 100 and to 200 °C, in the spectra of all the glasses, the structural bands related to vibrations of Pb–O/Sb–O (650–750 cm^–1^) are retained, and the intensity of the Si–O^–^ (970–990 cm^–1^) band exhibited the lowest intensity in the spectrum of Pb_30_ glass and increased when the content of Pb_3_O_4_ is raised from 30 to 35 mol %. When the temperature is raised to 300 °C, the Si–O^–^ band still exhibited the lowest intensity in the spectrum of Pb_30_ glass and the maximal intensity in the spectrum of Pb_35_ glass. The Raman spectral analysis revealed that the glass network remains structurally stable with increasing temperature up to 400 °C, particularly for compositions containing up to 30 mol % Pb_3_O_4_. In this range, the glassy core of SiO_2_ is largely preserved, indicating a robust and thermally stable structure. However, when the Pb_3_O_4_ content exceeds 30 mol %, notable changes in the local chemical environment were observed. Specifically, the spectrum of the Pb_35_ glass showed a pronounced increase in the intensity of the band associated with Si–O^–^ bond vibrations, suggesting a higher degree of depolymerization of the glass network. This indicates that beyond 30 mol % Pb_3_O_4_, the structural integrity of the glass begins to deteriorate, likely due to enhanced mobility of Pb^2+^ ions and reduced network connectivity. Overall, the extent of thermal transformation is strongly dependent on the Pb_3_O_4_ content: while glasses with 10–30 mol % Pb_3_O_4_ maintain stability up to 300 °C, those with higher Pb_3_O_4_ levels (such as Pb_35_) show early signs of structural reorganization.

### Dilatometry

Figure 3 shows the thermal expansion profiles obtained for the Pb_3_O_4_–Sb_2_O_3_–SiO_2_:Pr_2_O_3_ glasses were obtained with different concentrations of Pb_3_O_4_. The values of the linear coefficient of thermal expansion (CTE) were determined in the 50–100 °C, 50–200 °C, 50–300 °C, and 50–350 °C ranges and also dilatometric or softening temperature, *T*_s_ (the maximum registered temperature within the expansion region). The different CTE and *T*_s_ values obtained are summarized in Table 2. The CTE estimates obtained within the broad 50–350 °C range and the *T*_s_ values are also plotted in the insets of Figure 3 as a function of the Pb_3_O_4_ concentration in the glasses for a graphical appraisal. The results show the CTE first decreases while the dilatometric point increases from 10 to 30 mol % Pb_3_O_4_, with opposite behavior for 35 mol % Pb_3_O_4_, thus presenting an inflection point at 30 mol % Pb_3_O_4_. The data clearly shows that Pb_10_ glass exhibited the largest values of CTE with lowest *T*_s_. In other words, the magnitude of degree of polymerization of the glass network is gradually increased with an increase of the Pb_3_O_4_ content up to 30 mol % concentration, and for further increase, the depolymerization is more pronounced. This suggests that anharmonic/asymmetric vibrations of various bonds are more active in the Pb_35_ glass compared to that in other glasses.

**Figure 3:**
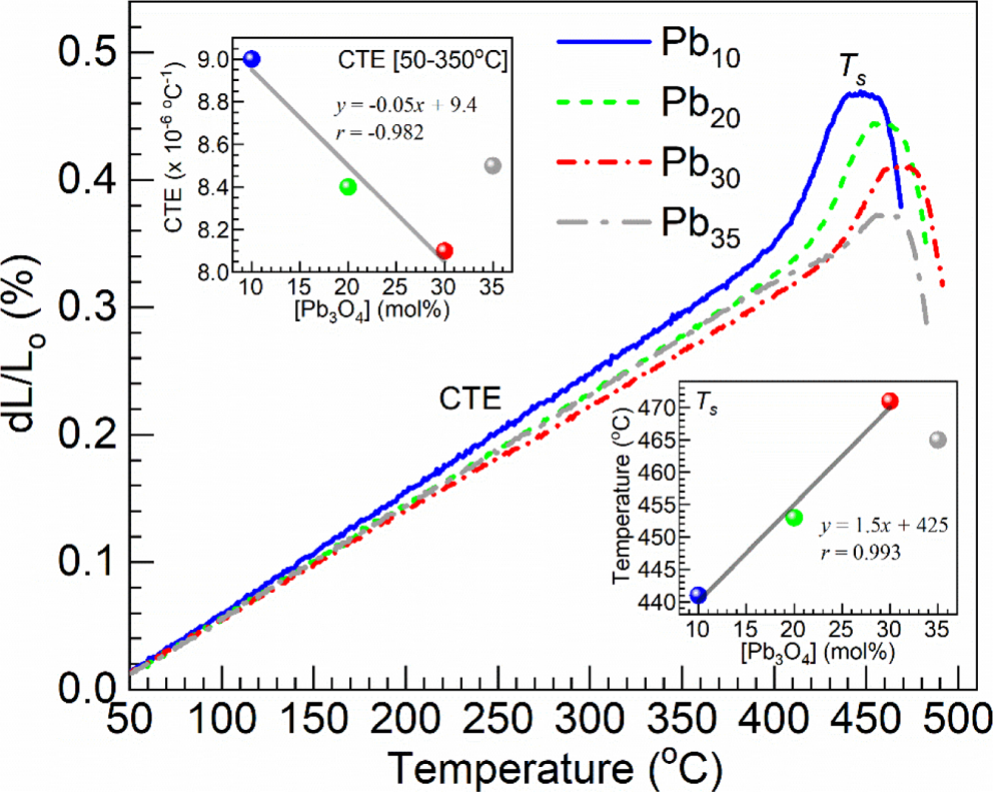
Dilatometric profiles obtained for the Pb_3_O_4_–Sb_2_O_3_–SiO_2_:Pr_2_O_3_ glasses with different concentrations of Pb_3_O_4_. The top and bottom insets are plots of the CTE (within 50–350 °C) and *T*_s_ values estimated, respectively, vs Pb_3_O_4_ concentration in the glasses; the solid lines are linear fits to the data for the samples with 10, 20, and 30 mol % Pb_3_O_4_ (equations and correlation coefficients, *r*, displayed).

**Table 2:** Summary of the Coefficients of Linear Thermal Expansion (CTE, Estimated in the Temperature Ranges of 50–100 °C, 50–200 °C, 50–300 °C, and 50–350 °C) and Dilatometric or Softening Temperature (*T*_s_) and Obtained for the Pb_3_O_4_–Sb_2_O_3_–SiO_2_/Pr_2_O_3_ Glasses

	CTE (×10^–6^ °C^–1^)				
glass	50–100 °C	50–200 °C	50–300 °C	50–350 °C	*T*_s_ (°C)
Pb_10_	8.2	8.9	8.9	9.0	441
Pb_20_	7.2	8.2	8.3	8.4	453
Pb_30_	7.6	8.1	7.9	8.1	471
Pb_35_	7.9	8.3	8.4	8.5	465

### Dielectric Properties and AC Conductivity

Thus far, Raman and thermal expansion studies clearly demonstrated a decreased degree of disorder in the glass network as the Pb_3_O_4_ content increased gradually from 10 to 30 mol % Pb_3_O_4_ and a larger degree of depolymerization in the Pb_35_ glass. Figure 4a illustrates the variation of the real part of the dielectric constant (ε′) of Pb_10_ glass with frequency across different temperatures, while Figure 4b depicts how ε′ changes with temperature at various frequencies. The data revealed that ε′ is notably higher at lower frequencies, especially at elevated temperatures. As mentioned earlier, PbO acts as a modifier and breaks the local symmetry of the glass network like any other modifier oxide. The resulting depolymerized glass network pave the way for the easy migration of free charge carriers and causes an increase in space charge polarization,^43,44^ a type of polarization that results from the accumulation of mobile charge carriers at the material interfaces particularly at the electrode surfaces. This is dependent on the concentration of the induced defects in the glass network. In glasses with a lower concentration of Pb_3_O_4_, these mobile charges respond to an external electric field but tend to become trapped near the electrodes due to interface boundaries. This accumulation leads to charge separation, contributing to an enhanced polarization effect, which in turn causes a significant rise in the dielectric constant at low frequencies.^13,44,45^

**Figure 4:**
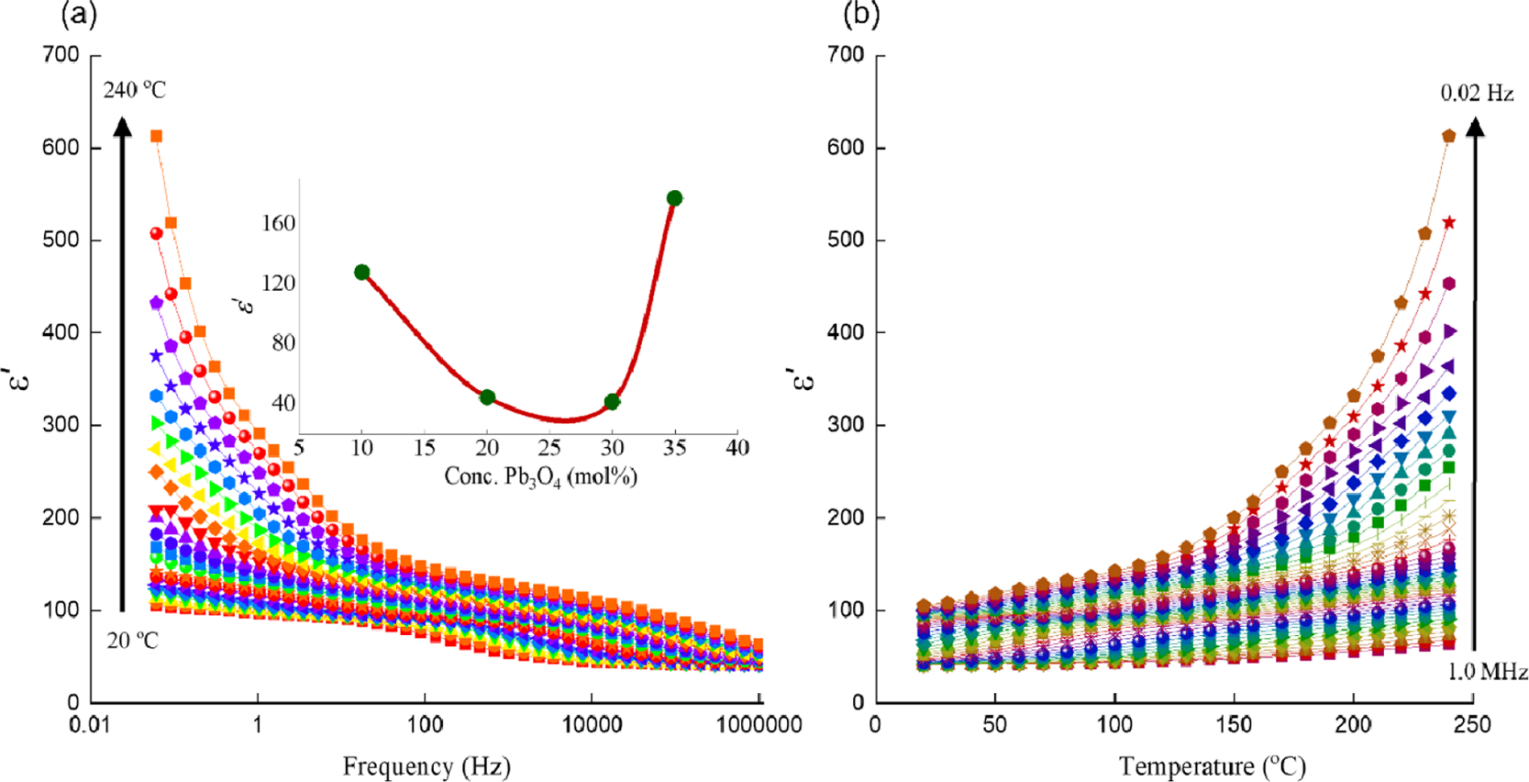
(a) Variation of the real part of the dielectric constant (ε′) of Pb_10_ glass with frequency across different temperatures. (b) Variation of ε′ with temperature at various frequencies for the same glass. The inset in panel (a) represents the variation of the dielectric constant with the concentration of Pb_3_O_4_ measured at 1 kHz and 200 °C.

In general, the effect of the temperature on the dielectric constant is quite complex. Typically, raising the temperature of glass materials leads to a noticeable reduction in the electronic component of the dielectric constant, especially over a temperature range of about 200 °C. Similarly, it appears that the ionic polarization component does not influence the dielectric constant to a significant change (up to about 10^11^ Hz) with such temperature variations. According to Debye’s theory, even the contribution from dipoles is expected to have minimal impact on the value of ε′ at such temperatures beyond the relaxation region. However, this study revealed a substantial increase in ε′, which is most likely due to enhanced space charge polarization. This can be attributed to the increased degree of depolymerization within the glass network.^46–48^ Among the glass samples examined, Pb_10_ exhibited the most pronounced rate of increase in ε′ at a given frequency. However, we observed a gradual decline in ε′ as the concentration of Pb_3_O_4_ increases beyond 10 mol % in the glass matrix (inset of Figure 4a). This trend indicates an increased degree of polymerization or an increased magnitude of interconnectivity among the structural units of the glass network, which restricts the mobility of charge carriers toward the electrodes. Consequently, the dielectric constant ε′ decreased with increasing Pb_3_O_4_ content up to 30 mol %. However, when the concentration of Pb_3_O_4_ is raised to 35 mol %, the dielectric constant exhibited a remarkable increase (inset of Figure 4a), especially at a higher temperature, indicating increasing degree of modifying action of lead ions. A similar conclusion could be drawn from the Raman spectra and thermal expansion studies, as discussed earlier.

In Figure 5a and b, the variations in ε″ (imaginary component of dielectric constant) with frequency and temperature for Pb_20_ glass are presented. The variations are found to be similar to those of the real component of the dielectric constant. It may be noted here that the plots in Figures 4 and 5 have not exhibited any considerable relaxation effects probably due to the masking of such effects by electrodes. Hence, to gain deeper insight into the dipolar behavior of the glass, we adopted the electric moduli (*M*′ and *M*″) formalism because the variation of such coefficients either with frequency or with temperature reasonably eliminates the influence of electrode phenomena on relaxation processes.

**Figure 5:**
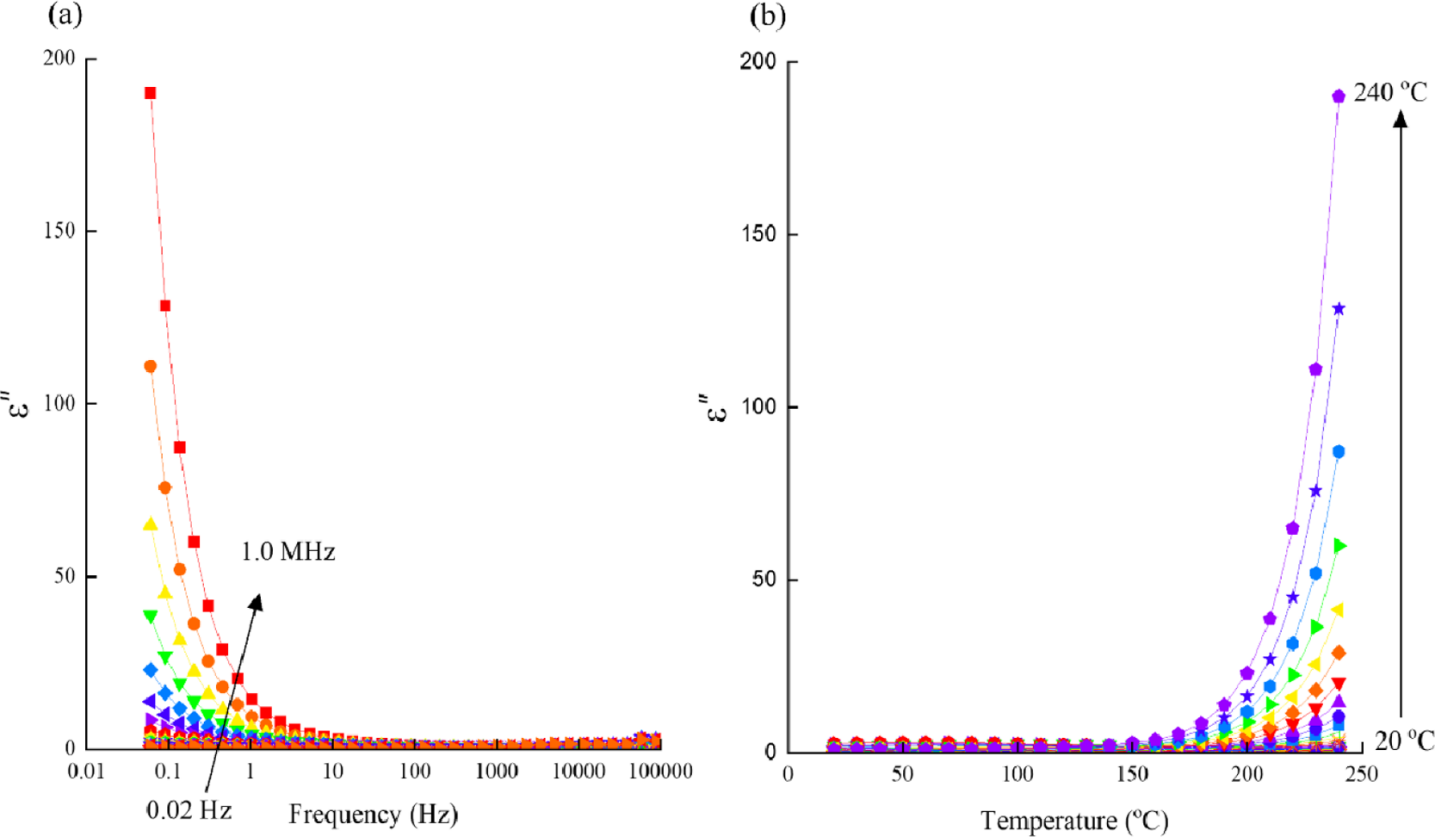
Variation of ε″ (a) with frequency and (b) with temperature for the Pb_20_ glass.

Using real and imaginary parts of the dielectric constant, the real (*M*′) and imaginary (*M*″) coefficients of electric moduli are evaluated using the components of the dielectric constant with the following equations

1 $$M^{\prime }=\frac{\varepsilon^{\prime }\left(\omega \right)}{{\left(\varepsilon^{\prime }\left(\omega \right)\right)}^{2}+{\left(\varepsilon^{\prime \prime }\left(\omega \right)\right)}^{2}}$$

and

2 $$M^{\prime \prime }\left(\omega \right)=\frac{\varepsilon^{\prime \prime }\left(\omega \right)}{{\left(\varepsilon^{\prime }\left(\omega \right)\right)}^{2}+{\left(\varepsilon^{\prime \prime }\left(\omega \right)\right)}^{2}}$$

The variations in *M*′ and *M*″ with the frequency and temperature of Pb_10_ glass are illustrated in Figure 6a and b, respectively. These plots clearly exhibited dipolar relaxation phenomena. In the *M*″ versus frequency curves, the initial segment corresponds to the oscillations of dipoles with larger amplitudes, while the latter portion represents the regime where dipole oscillations are confined within potential wells, limiting their displacement. Using these graphs, the relaxation time (τ) at different temperatures is estimated and presented in Table 3. Using the plots of ln­(τ) vs 1/*T* (Arrhenius plots), the activation energy (*W*_d_) for dipolar relaxation is calculated and summarized in Table 3. The results show that the activation energy increased progressively with increasing Pb_3_O_4_ content up to 30 mol %. This suggests a reduction in the ability of dipoles to reorient in response to the external electric field, implying that higher Pb_3_O_4_ concentrations make the glass network more rigid and less accommodating to dipolar oscillations. However, a decrease in the activation energy was observed for glass Pb_35_ (with respect to that of Pb_30_). This observation is consistent with the results of the Raman spectral studies. In glasses containing Sb_2_O_3_, antimony oxide is known to integrate into the glass network in the form of SbO_3_ pyramidal units that possess a lone pair of electrons at the pyramid apex. These structural units have been reported to possess a net-dipole moment, which plays a significant role in the observed dipolar relaxation phenomena.^49^

**Figure 6:**
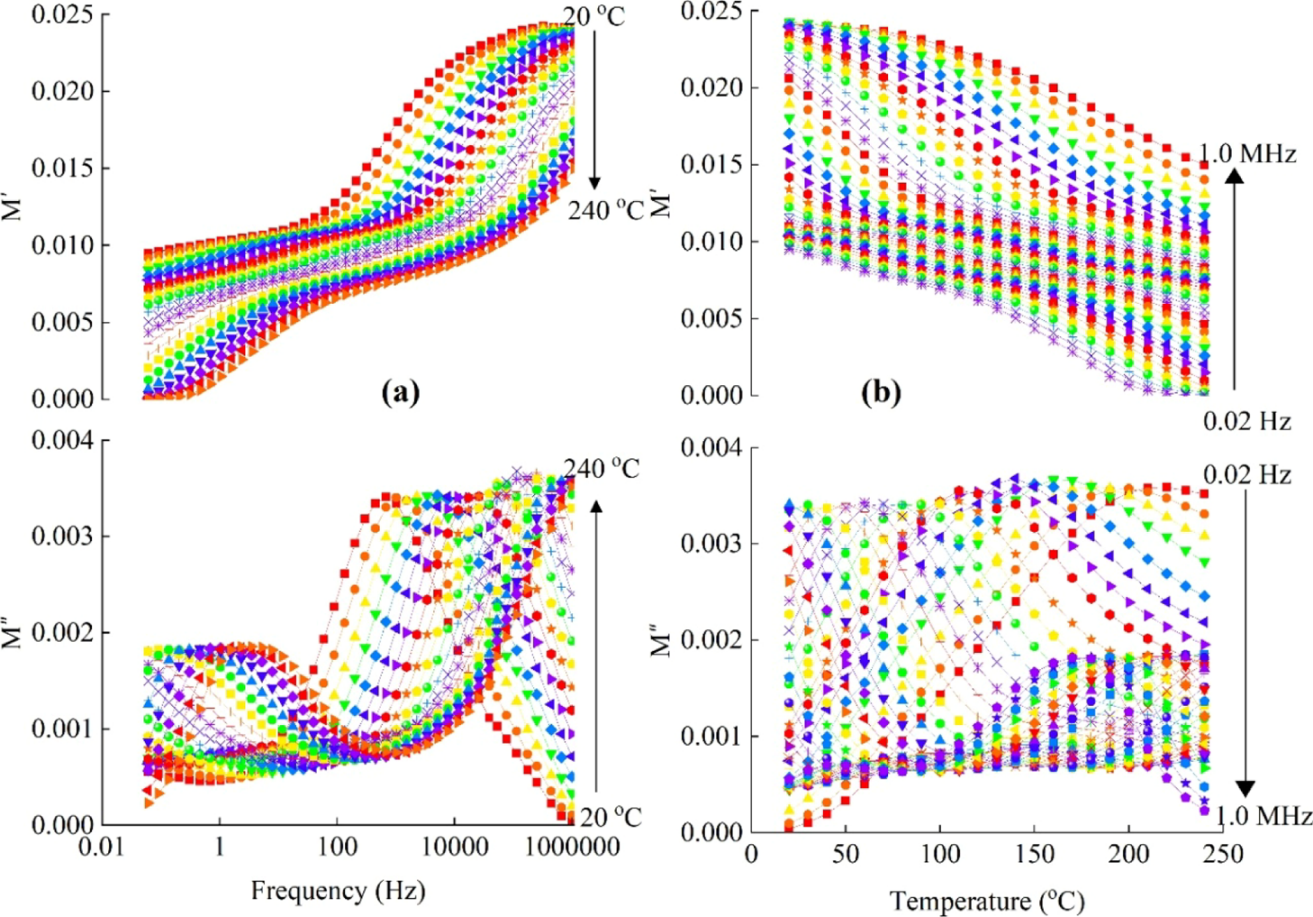
Variations of *M*′ (top panels) and *M*″ (bottom panels) with (a) frequency and (b) temperature for Pb_10_ glass.

**Table 3:** Activation Energy (A.E.) and Key Dielectric Parameters of Pr^3+^-Doped Pb_3_O_4_–Sb_2_O_3_–SiO_2_ Glasses

glass	A.E. for conduction, *W*_ac_ (eV)	relaxation time, τ_M_ (ms) at 240 °C	A.E. for dipoles, *W*_d_ (eV)	spreading factor α (rads)
Pb_10_	0.295	45.0	0.212	0.396
Pb_20_	0.312	78.4	0.283	0.352
Pb_30_	0.354	272	0.425	0.262
Pb_35_	0.315	120	0.242	0.323

To elaborate further, Cole–Cole plots (*M*″ vs *M*′) across the temperature range of 200–240 °C are constructed and illustrated in Figure 7a–d for all the studied glasses. These plots exhibit semicircular arcs, with centers positioned below the *x*-axis. The nondispersion of these curves in this temperature range indicates the relaxation time is independent of temperature. An angle, α, subtended between the *x*-axis and the line connecting the arc centers to the origin, is clearly visible. The presence of a nonzero α signifies the distribution of dipolar relaxation times (τ). Among the samples, Pb_10_ glass (refer to Table 3) was found to have the largest α value, indicating a broader dispersion of relaxation times. A nonzero α typically points to the existence of multiple relaxation processes or various dipoles possessing distinct dipole moments.^50^ The broad distribution of relaxation times may result from the interaction between individual relaxation processes, where the relaxation of one site is dependent on the prior relaxation of the other.

**Figure 7:**
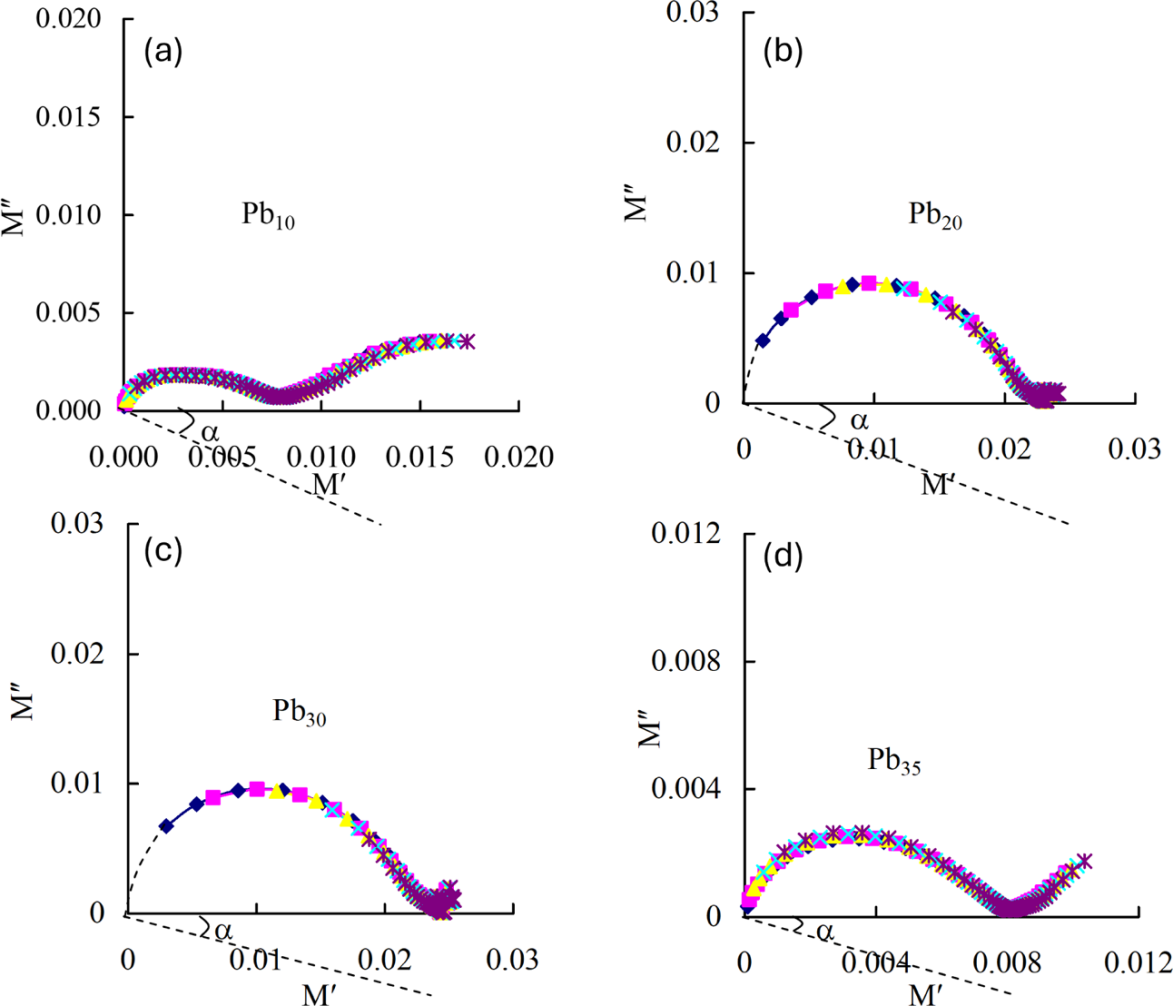
Cole–Cole plots (*M*″ vs *M*′) drawn in the temperature range of 200–240 °C for the Pb_3_O_4_–Sb_2_O_3_–SiO_2_:Pr_2_O_3_ glasses mixed with different concentrations of Pb_3_O_4_: (a) Pb_10_; (b) Pb_20_; (c) Pb_30_; and (d) Pb_35_.

Even when all sites share an identical relaxation time (τ), their mutual coupling causes an effective extension in the time domain, thereby leading to the observed spread in relaxation behavior.^51^ Furthermore, even identical dipoles embedded in different local potential environments can contribute to relaxation time distribution, leading to a broader spread of relaxation behavior.^52^

Assuming that the effective electric field within these glasses behaves as a Lorentz field, the relationship between dipole density *N* (number of dipoles per unit volume), dipole moment μ, and dielectric constants at low and high frequencies (ε_s_ and ε_0_, respectively) can be described using the modified Clausius–Mossotti–Debye relation:^50^

3 $$\frac{\varepsilon_{s}-1}{\varepsilon_{s}+2}-\frac{\varepsilon_{0}-1}{\varepsilon_{0}+2}=\frac{4\pi N\mu^{2}}{9kT}$$

On rearranging the terms, we get

4 $$\frac{\varepsilon_{s}-\varepsilon_{0}}{\left(\varepsilon_{s}+2\right)\left(\varepsilon_{0}+2\right)}=\frac{4\pi N\mu^{2}}{27k}$$

Converting this into electric moduli formalism, we get

5 $$\frac{4\pi N\mu^{2}}{3K}=\frac{M_{0}-M_{s}}{\left(1+2M_{0}\right)\left(1+2M_{s}\right)}T$$

where $M_{s}=\frac{1}{\epsilon_{s}}\;\text{and}\;M_{0}=\frac{1}{\epsilon_{0}}$ . Here, *M*_s_ and *M*_0_ represent the electric moduli at the lower and higher frequencies, respectively. Since, in the studied glass samples, the ions and dipoles within the glass network can be reasonably approximated as point entities and their concentration is not excessively high, the applicability of the eq 5 remains valid for these systems. The term *N*μ^2^ on the right side of eq 4 indicates the collective strength of dipoles. By substitution of the values of *M*_s_ and *M*_0_ into eq 5, 4π*N*μ^2^/27*k* is evaluated at *T* = 513 K for various concentrations of Pb_3_O_4_. The variation of this quantity with Pb_3_O_4_ content exhibited a nonlinear behavior, as represented in Figure 8. Such behavior of the plot confirms the spreading of relaxation times or the existence of dipoles possessing different dipole moments within the studied glass matrices.

**Figure 8:**
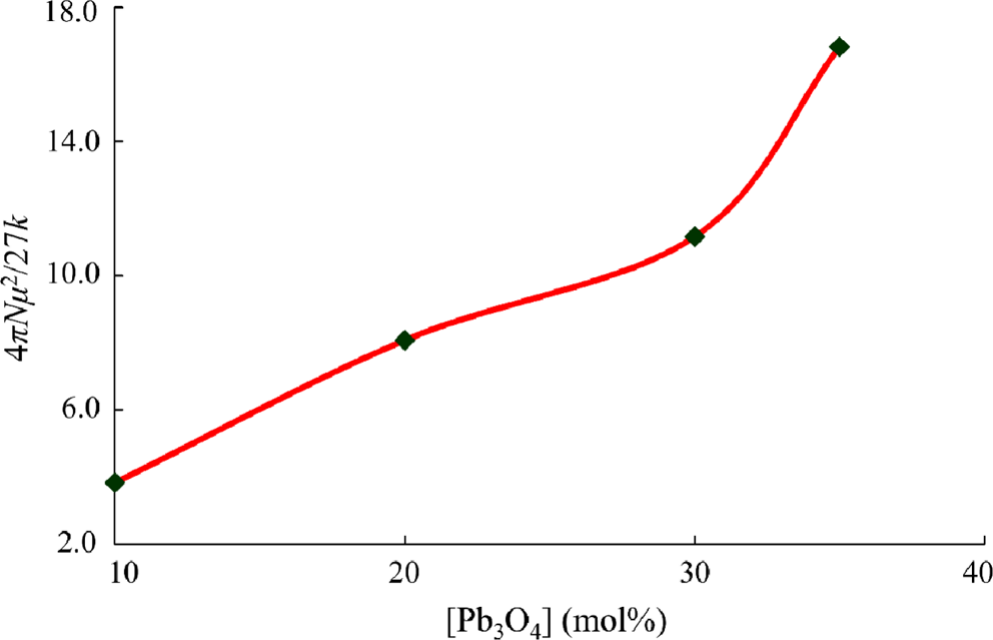
Variation of the quantity 4π*N*μ^2^/27*k* evaluated at *T* = 513 K with Pb_3_O_4_ content.

Figure 9a and b illustrate how frequency affects the real and imaginary components of the impedance of Pb_35_ glass at different temperatures. The real part (*Z*′), which represents pure resistance, shows an almost linear decrease with frequency. On the other hand, the imaginary part (*Z*″) increases linearly with frequency up to approximately 1 kHz, indicating the presence of inductive reactance (*L*ω). Beyond this frequency, *Z*″ begins to decrease inversely with frequency, demonstrating a capacitive behavior characterized by (1/*C*ω). A similar frequency-dependent impedance pattern is observed in the other glass samples, as well.

**Figure 9:**
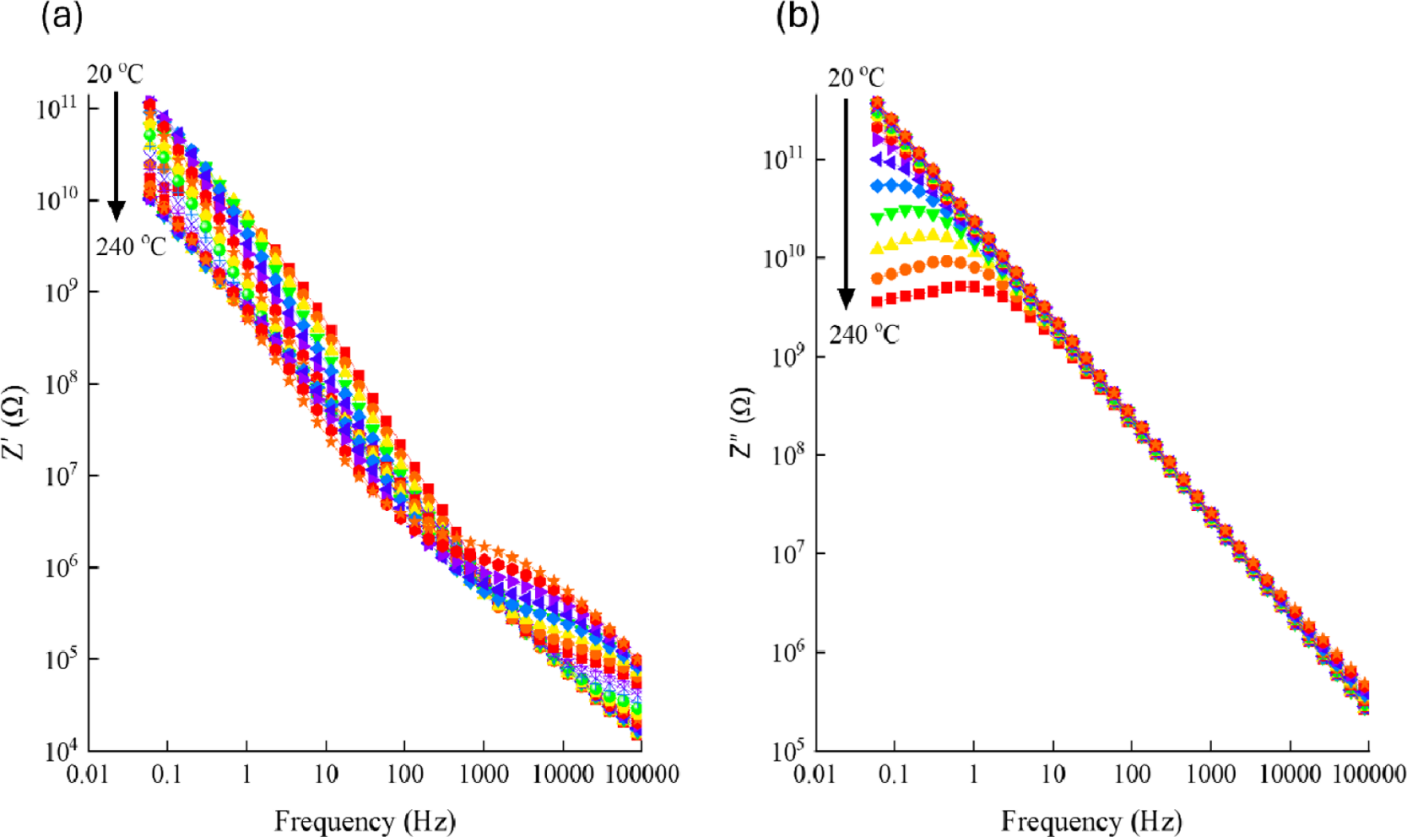
Variation of (a) real and (b) imaginary components of the impedance of Pb_35_ glass with frequency measured at different temperatures.

To further analyze the impact of temperature and Pb_3_O_4_ content on impedance magnitude, Nyquist plots (*Z*″ vs *Z*′) were generated for Pb_10_ glass at various temperatures, as shown in Figure 10a,b. With increasing temperature, the area enclosed by these curves diminishes significantly, indicating a reduction in impedance with temperature.

**Figure 10:**
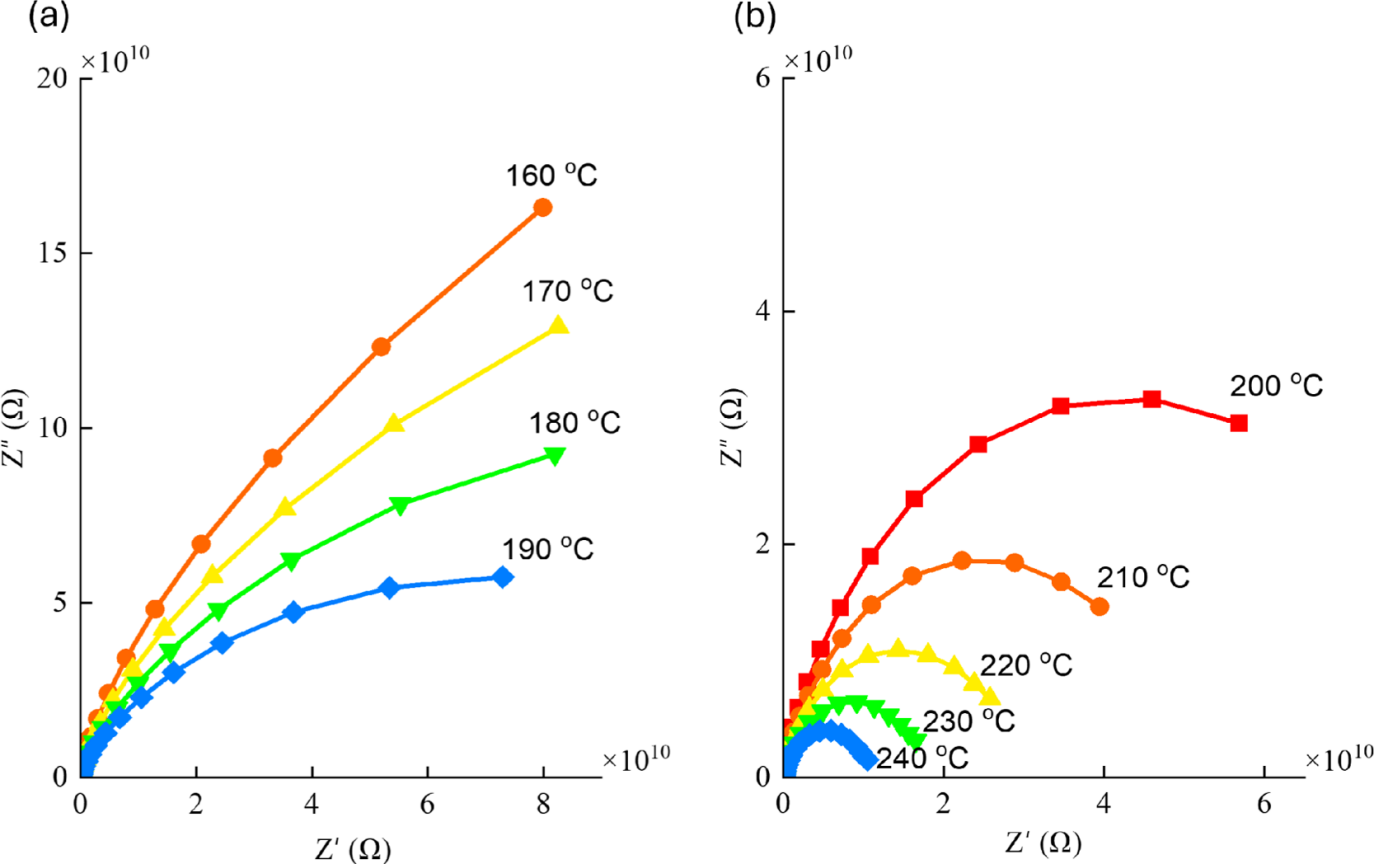
Nyquist plots (*Z*″ vs *Z*′) generated for Pb_10_ glass at various temperatures: (a) 160–190 °C and (b) 200–240 °C.

A comparative study of impedance across all glass samples at 240 °C is depicted in Figure 11. This comparison revealed that the area under the Nyquist plots increases progressively with Pb_3_O_4_ content up to 30 mol %, suggesting that impedance increases as the Pb_3_O_4_ concentration rises to this concentration. When the content of Pb_3_O_4_ is raised from 30 to 35 mol %, a decrement in the area enclosed by the curve is noticed. This trend is consistent with earlier findings, where a higher Pb_3_O_4_ content up to 30 mol % was found to enhance the degree of polymerization in the glass network, thereby increasing its electrical resistivity, and for further increase, a decrement in the impedance is possible as mentioned in the Raman spectral analysis. It may be noted here that a similar set of plots for γ-MnO_2_ on lead anodes was presented by Minakshi et al.^53^ Their study demonstrated that, in the low-frequency region, the Nyquist plots exhibited a near-vertical line approaching 90°, thereby evidencing enhanced ion transport properties and improved electrodeposition behavior.^53^

**Figure 11:**
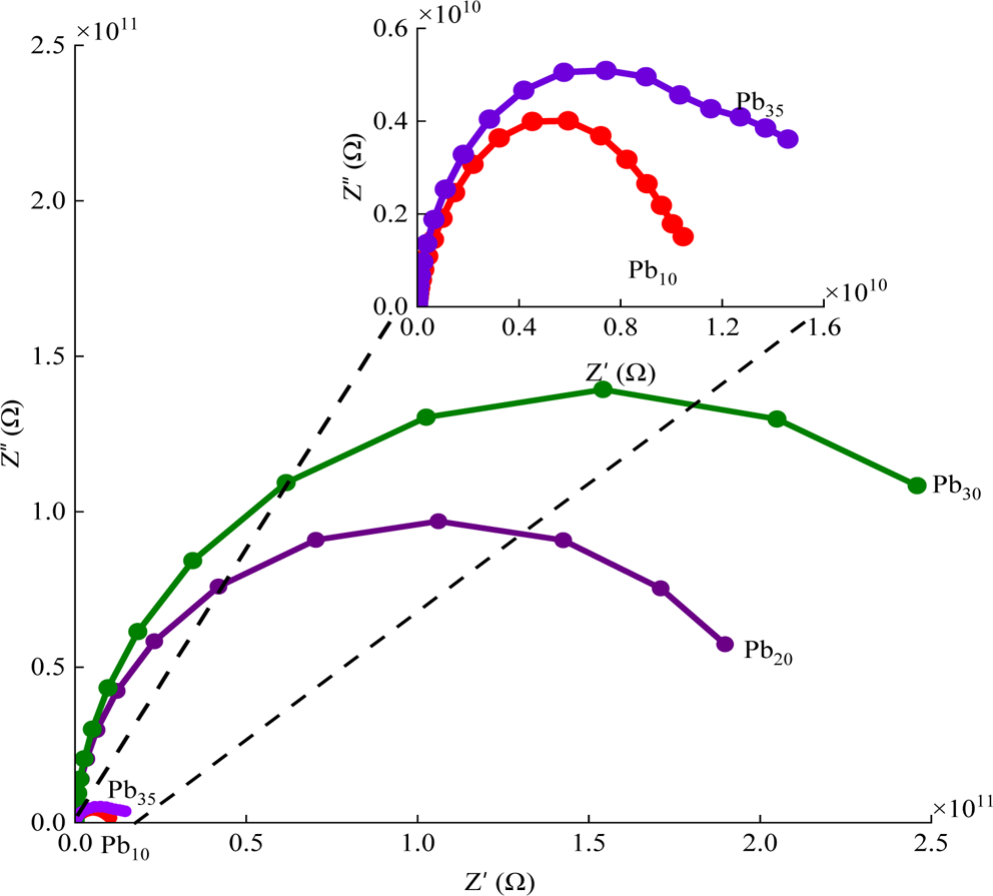
Comparison of impedance for the Pb_3_O_4_–Sb_2_O_3_–SiO_2_:Pr_2_O_3_ glasses mixed with different concentrations of Pb_3_O_4_ at 240 °C.

Figure 12a and b illustrate the variations in ac conductivity (σ_ac_) of Pb_35_ glass as functions of frequency and reciprocal temperature (1/*T*), respectively. At lower frequencies, σ_ac_ displayed a linear dependence on 1/*T*, while at higher frequencies and lower temperatures, the conductivity appeared to be nearly independent of temperature. Similar behavior was observed for the other samples as well. The inset of Figure 12a shows the dependence of σ_ac_ on the Pb_3_O_4_ content, where a gradual decline in conductivity was noted as the Pb_3_O_4_ concentration increased from 10 to 30 mol % and a slight increase was observed beyond this concentration.

**Figure 12:**
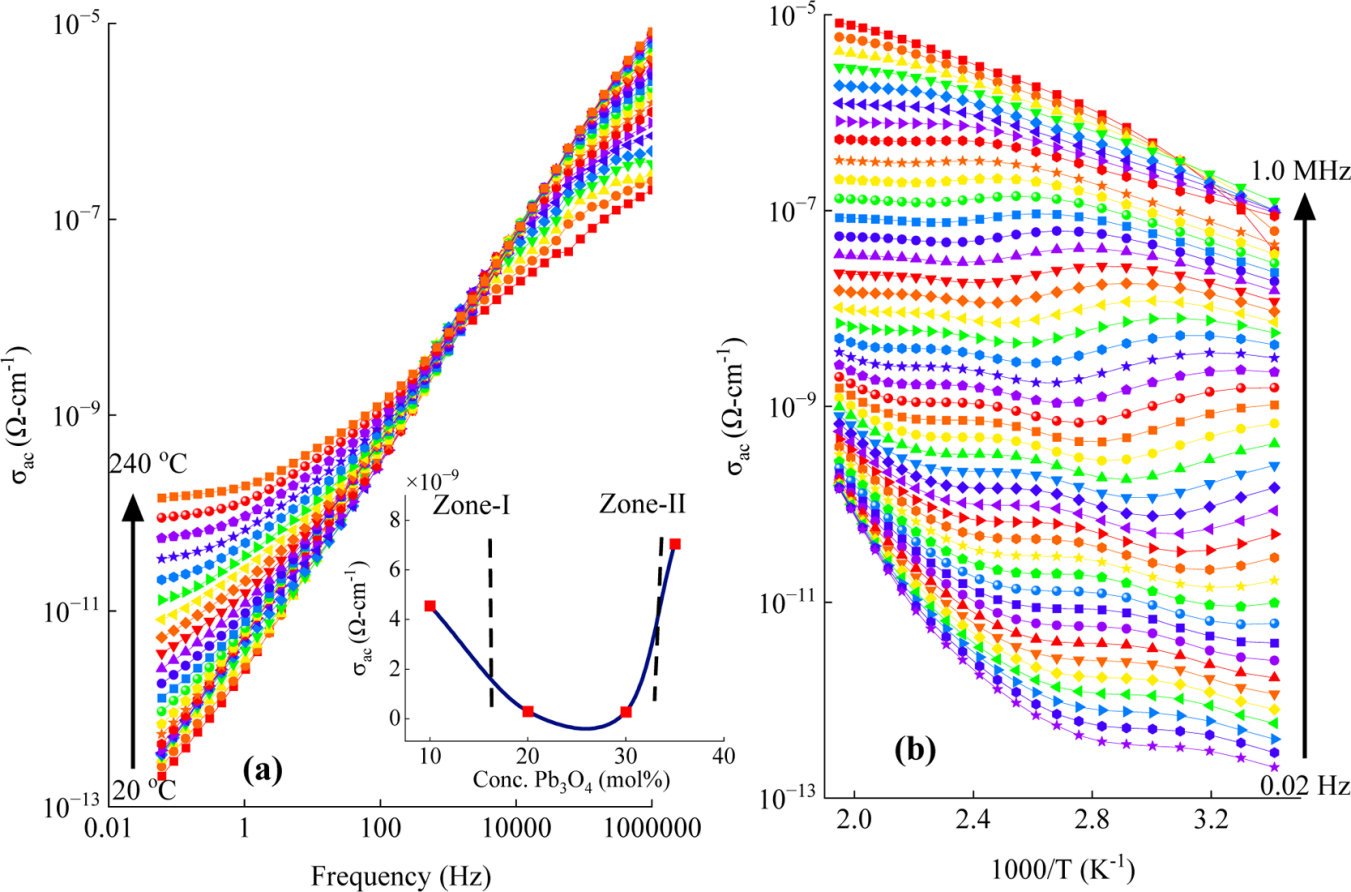
Variations in ac conductivity (σ_ac_) of the Pb_35_ glass with (a) frequency and (b) reciprocal temperature (1/*T*). The inset of (a) shows the dependence of σ_ac_ on the Pb_3_O_4_ content measured at 1 kHz and 200 °C.

The activation energy for ac conduction (*W*_ac_) was determined from the slopes of the log σ_ac_ versus 1/*T* plots in the higher temperature range. The value of *W*_ac_ is found to be the largest for the Pb_30_ glass and decreased beyond 30 mol % Pb_3_O_4_, as shown in Table 3. This trend implies a strengthening of the internal structure of the glass network (up to 30 mol % of Pb_3_O_4_), which increasingly restricts the mobility of charge carriers. As previously discussed, increasing the Pb_3_O_4_ content from 10 to 30 mol % leads to a more polymerized and thermally stable glass network. This stability is attributed to the strong and interconnected SiO_2_-based framework, which effectively limits the mobility of charge carriers. Consequently, electrical conductivity decreases within this concentration range due to reduced ionic movement and fewer available pathways for charge transport. However, at 35 mol % Pb_3_O_4_, the glass begins to exhibit signs of depolymerization, as evidenced by a noticeable decline in the intensities of Si–O and PbO_4_ vibrational bands in the Raman spectra and pronounced structural degradation at elevated temperatures. These changes point to a disruption in the network connectivity and an increase in the mobility of Pb^2+^ ions, indicating a transition from a robust, thermally stable matrix to a more disordered and thermally sensitive structure. This shift results in a significant increase in electrical conductivity as the Pb_3_O_4_ content rises from 30 to 35 mol %.

In the composition range of 10–30 mol % (Zone 1), the compact and polymerized glass network restricts ionic mobility but the presence of mixed-valence Pb^2+^/Pb^4+^ and Sb^3+^/Sb^5+^ ions supports small polaron conduction through electron hopping. Due to the limited number of hopping sites and the rigid structure, polaronic conduction is the dominant mechanism in this range, although the overall conductivity remains low. At 35 mol % Pb_3_O_4_ (Zone 2), the network undergoes depolymerization with the formation of more nonbridging oxygens and increased structural disorder. These changes create greater free volume and conduction channels, facilitating the enhanced mobility of Pb^2+^ ions. While polaron hopping may still contribute due to continued redox activity and Pb clustering, ionic conduction becomes the predominant transport mechanism. This transition accounts for the marked increase in electrical conductivity observed in this higher concentration range. This mechanism suggests that glasses containing 30–35 mol % Pb_3_O_4_ are suitable for use as solid-state electrolytes in batteries. However, Pb^2+^ ions are larger in size and heavy, with low mobility compared to those of Li^+^ and Na^+^ ions. Nevertheless, this mechanism is useful for fundamental understanding and may inspire the design of analogous glass systems doped with mobile alkali ions (e.g., Li^+^, Na^+^) for practical battery applications.

## Conclusions

Raman spectroscopy results combined with dilatometric studies indicated that increasing of Pb_3_O_4_ from 10 to 30 mol % in a Sb_2_O_3_–SiO_2_:Pr_2_O_3_ glass leads to progressive polymerization of the glass network, resulting in a compact and rigid structure. Beyond 30 mol % (at 35 mol %), the glass network underwent depolymerization, forming more nonbridging oxygens (NBOs) and increasing structural disorder. The dilatometric analysis harmonized with the structural appraisal as it revealed that the thermal expansion coefficients first decreased from 10 to 30 mol % Pb_3_O_4_, indicating an increase in glass rigidity in this regime, followed by an increase the CTE for 35 mol % Pb_3_O_4_. On the other hand, the softening temperatures first increased from 10 to 30 mol % Pb_3_O_4_, implying a glass strengthening effect, but then decreased at 35 mol %, characterizing the looser structure indicated to be depolymerized relative to 30 mol % Pb_3_O_4_. The dielectric constant and ac conductivity decreased with increasing Pb_3_O_4_ up to 30 mol % due to restricted ionic mobility in the compact structure. Conduction in this range is primarily through small polaron hopping facilitated by the mixed valence states of Pb^2+^/Pb^4+^ and Sb^3+^/Sb^5+^. At 35 mol % Pb_3_O_4_, ionic conduction dominates due to network depolymerization, enhanced free volume, and formation of conduction channels. This shift significantly increases electrical conductivity, indicating a transition from polaronic to ionic transport. Glasses with 30–35 mol % Pb_3_O_4_ show promise as solid-state electrolytes due to improved conduction characteristics. Despite Pb^2+^’s low mobility compared to alkali ions, the insights gained are valuable for designing future alkali-doped glass systems (e.g., with Li^+^ or Na^+^) for practical battery applications.

## References

[1] Kumari C., Chhoker S., Sharma P. (2023). Effect of rare earth dopant on the
ac conductivity and dielectric study of GeSbSe chalcogenides glasses. J. Non-Cryst. Solids.

[2] Zaki A. A., Sheha E., Farrag M., Salman F. (2022). Study of ionic conduction,
dielectric relaxation, optical and electrochemical properties of AgPO_3_/graphene glasses for magnesium battery applications. J. Non-Cryst. Solids.

[3] Assad H., Kharroubi M. (2021). Dielectric
studies and Cole-Cole plot analysis of Na_2_O–(1–x)­ZnO–xCoO–P_2_O_5_ glasses. J. Non-Cryst.
Solids.

[4] Liu Y., Liang T., Zheng W., Liu Y., Fu H., Liu Q., Wu C., Zhang J., Chen H., Gao L., Chen D., Li Y. (2025). Effects of
Al_2_O_3_ on the coefficient of thermal expansion
and dielectric properties
of borosilicate glasses as an interposer for 3D packaging. Ceram. Int..

[5] Ravi
Kumar G., Koteswara Rao M., Srikumar T., Rao M. C., Ravi Kumar V., Veeraiah N., Rao C. S. (2018). Spectroscopic, dielectric
dispersion and dc conductivity studies of Sb_2_O_3_ doped lithium fluoro borophosphate glasses mixed with small concentrations
of NiO. J. Alloys Compd..

[6] Vinothkumar P., Dhavamurthy M., Mohapatra M., Murugasen P. (2021). Structural,
optical and thermo-physical characterizations of co-doped Pr^3+^ and Nd^3+^ ions on BaCO_3_–H_3_BO_3_ glasses for microelectronic applications. Bull. Mater. Sci..

[7] Li S., Lu Y., Qu Y., Kang J., Yue Y., Liang X. (2021). Dielectric
and thermal properties of aluminoborosilicate glasses doped with mixed
rare-earth oxides. J. Non-Cryst. Solids.

[8] Arunachalam S., Kirubasankar B., Pan D., Liu H., Yan C., Guo Z., Angaiah S. (2020). Research progress
in rare earths and their composites
based electrode materials for supercapacitors. Green Energy Environ..

[9] Kalužný J., Pedlíková J., Kostka P., Labas V., Kubliha M., Zavadil J., Minárik S. (2009). Investigation
of electrical and dielectric properties of Ge_20_Se­(80–x)­Te_x_ glasses doped by Er,Ho,Pr. J. Optoelectron.
Adv. Mater..

[10] Anjaiah J., Laxmikanth C., Mwanga S. F., Raju P., Mohammad
Ali S. K., Shankar J., Neeraja Rani G., Mwankemwa B. (2022). Influence of rare-earth ion doping on dielectric properties
of lithium zinc borate glasses. Opt. Mater..

[11] Ismail M. M., Abo-Mosallam H. A., Darwish A. G. (2025). Synthesis, mechanical,
and dielectric
properties of BaO–CdO–PbO–CeO_2_–B_2_O_3_ glass system through Sm_2_O_3_ doping for advanced dielectric applications. Ceram. Int..

[12] Blanc W., Mauroy V., Nguyen L., Shivakiran Bhaktha B. N., Sebbah P., Pal B. P., Dussardier B. (2011). Fabrication
of rare earth-doped transparent glass ceramic optical fibers by modified
chemical vapor deposition. J. Am. Ceram. Soc..

[13] Kumar P. A., Kostrzewa M., Ingram A., Baskaran G. S., Venkatramaiah N., Venkatramu V., Kumar V. R., Veeraiah N. (2025). Impact of Ag_2_O doping on the structural and conductive features of Na_2_O–SiO_2_–P_2_O_5_–Y_2_O_3_ glass ceramics embedded with Na_2_AgY­(Si_2_O_5_)_3_ crystallites for applications as
solid-state electrolytes. J. Alloys Compd..

[14] Vijayakrishna S., Pavić L., Bafti A., Pisk J., Bhadrarao D., Dana Rao Y., Venkata Sekhar A., Chitti Babu V., Ravi Kumar V., Veeraiah N. (2024). Impact of Cr^3+^/Mo^6+^/W^6+^ doping on dipolar relaxation and
AC conductivity
in Li_2_O–Al_2_O_3_–SiO_2_ glasses. Phys. Status Solidi A.

[15] Pavić L., Narasimha Rao N., Moguš-Milanković A., Šantić A., Ravi Kumar V., Piasecki M., Kityk I. V., Veeraiah N. (2014). Physical properties of ZnF_2_–PbO–TeO_2_:TiO_2_ glass ceramics – Part III dielectric
dispersion and ac conduction phenomena. Ceram.
Int..

[16] Yang M., Chen C., Yang R., Zu Q., Huang S., Zhang Y., Zeng H. (2025). Effect of phosphorus
on the structural
nonhomogeneity and dielectric properties of alkaline earth aluminoborosilicate
glasses. J. Non-Cryst. Solids.

[17] Churbanov M. F., Denker B. I., Galagan B. I., Koltashev V. V., Plotnichenko V. G., Snopatin G. E., Sukhanov M. V., Sverchkov S. E., Velmuzhov A. P. (2021). Laser potential of Pr^3+^ doped chalcogenide
glass in 5–6 μm spectral range. J. Non-Cryst. Solids.

[18] Suresh B., Purnachand N., Zhydachevskii Y., Brik M. G., Reddy M. S., Suchocki A., Piasecki M., Veeraiah N. (2017). Influence of Bi^3+^ ions
on the amplification of 1.3 μm emission of Pr^3+^ ions
in lead silicate glasses for the applications in second
telecom window communications. J. Lumin..

[19] Shen X., Zhang Y., Xia L., Li J., Yang G., Zhou Y. (2020). Dual super-broadband NIR emissions
in Pr^3+^–Er^3+^–Nd^3+^ tri-doped
tellurite glass. Ceram. Int..

[20] Shoaib M., Khan I., Rooh G., Wabaidur S. M., Islam M. A., Chanthima N., Kothan S., Ullah I., Ahad A., Kaewkhao J. (2022). Judd-Ofelt
and luminescence properties of Pr^3+^ doped ZnO–Gd_2_O_3_/GdF_3_–BaO–P_2_O_3_ glasses for visible and NIR applications. J. Lumin..

[21] Sudhakar P., Siva Sesha Reddy A., Zhydachevsky Y., Brik M. G., Suchocki A., Ravi Kumar V., Piasecki M., Veeraiah N. (2019). Influence of some thermally
resistant transition metal oxides on emission features of Pr^3+^ ions in zinc borate glasses. J. Non-Cryst.
Solids.

[22] Mokhtar K., Mohamed K., Lakhdar G., Sébastien B., Hamza A. (2021). Electrical conductivity and dielectric
properties of rare earth ions
(Ce^3+^, Pr^3+^ and Eu^3+^) doped in zinc
sodium phosphate glass. J. Non-Cryst. Solids.

[23] El-Shamy N. T., Mahrous E. M., Alghamdi S. K., Tommalieh M. J., Rabiea E. A., Abomostafa H. M., Abulyazied D. E., Abouhaswa A. S. (2024). Influence of Pr^3+^ ions
on structural, photoluminescence,
dielectric, and mechanical properties of barium lithium fluoroborate
glasses. Inorg. Chem. Commun..

[24] Kubliha M., Trnovcová V., Furár I., Kadlečíková M., Pedlíková J., Greguš J. (2009). Structural
peculiarities, and electrical and optical properties of 70TeO_2_·30PbCl_2_ glasses doped with Pr^3+^, prepared in Pt or Au crucibles. J. Non-Cryst.
Solids.

[25] Guo D., Robinson C., Herrera J. E. (2016). Mechanism
of dissolution of minium
(Pb_3_O_4_) in water under depleting chlorine conditions. Corros. Sci..

[26] Zhang H., Liu S. H., Liu F., Yan S. L., Li W. Y. (2018). Study on
the reaction mechanism between Pb_3_O_4_ and Si
in stored silicon delay composition. J. Therm.
Anal. Calorim..

[27] Kut T. V. N. K., Bafti A., Pisk J., Pavić L., Sekhar A. V., Naresh P., Reddy A. S. S., Raju G. N., Kumar V. R., Veeraiah N. (2023). Dielectric features
of Au_2_O_3_ doped Li_2_O–SiO_2_ glass
system-influence of Pb_3_O_4_. J. Non-Cryst. Solids.

[28] Bhadrarao D., Brik M. G., Pavić L., Bafti A., Pisk J., Sekhar A. V., Venkatramaih N., Kumar V. R., Raju G. N., Veeraiah N. (2025). Structural and optoelectronic
potential of Ag_2_BiO_3_-embedded red lead silver-bismuth
borate glass-ceramics. J. Mol. Struct..

[29] Rao Y. D., Venkatramaiah N., Sekhar A. V., Purnachand N., Kumar V. R., Veeraiah N. (2023). Impact of
red lead on 0.65 and 1.3
μm emissions of Pr^3+^ ions in a non-conventional antimony
oxide glass system for application in optical communication. J. Mater. Sci.: Mater. Electron..

[30] Ashok J., Kostrzewa M., Srinivasa Reddy M., Ravi Kumar V., Venkatramiah N., Piasecki M., Veeraiah N. (2019). Structural and physical
characteristics of Au_2_O_3_ doped sodium antimonate
glasses – part I. J. Am. Ceram. Soc..

[31] Capeletti, L. B. ; Zimnoch, J. H. Fourier Transform Infrared and Raman Characterization of Silica-Based Materials. In Applications of Molecular Spectroscopy to Current Research in the Chemical and Biological Sciences; Stauffer, M. T. , Ed.; InTechOpen: London, UK, 2016.

[32] Nagaraju R., Ramadevudu G., Haritha L., Kumar N. P. (2024). Physical,
optical,
spectroscopic features of Li_2_B_4_O_7_–Bi_2_O_3_–Sb_2_O_3_ glass system reinforced with molybdenum ions. Ceram. Int..

[33] Refaat A., Ibrahim M. A., Shehata D., Elhaes H., Ibrahim A., Mamatkulov K., Arzumanyan G. (2024). Design, characterization and implementation
of cost-effective sodium alginate/water hyacinth microspheres for
remediation of lead and cadmium from wastewater. Int. J. Biol. Macromol..

[34] Rao, K. J. Structural Chemistry of Glasses; Elsevier: Amsterdam, The Netherlands, 2002.

[35] Sendova M., Jiménez J. A., Honama C. (2016). Rare earth-dependent
trend of the
glass transition activation energy of doped phosphate glasses: Calorimetric
analysis. J. Non-Cryst. Solids.

[36] Sendova M., Jiménez J. A. (2018). Synergistic
thermo-Raman and calorimetric kinetic study
of the cation modifier’s role in binary metaphosphate glasses. J. Raman Spectrosc..

[37] Guerette M., Huang L. (2015). In-situ Raman and Brillouin
light scattering study of the international
simple glass in response to temperature and pressure. J. Non-Cryst. Solids.

[38] Feller S., Lodden G., Riley A., Edwards T., Croskrey J., Schue A., Liss D., Stentz D., Blair S., Kelley M., Smith G., Singleton S., Affatigato M., Holland D., Smith M. E., Kamitsos E. I., Varsamis C. P. E., Ioannou E. (2010). A multispectroscopic
structural study
of lead silicate glasses over an extended range of compositions. J. Non-Cryst. Solids.

[39] Jia H., Chen G., Wang W. (2006). UV irradiation-induced
Raman spectra
changes in lead silicate glasses. Opt. Mater..

[40] McMillan P., Piriou B. (1983). Raman spectroscopic
studies of silicate and related
glass structure: a review. Bull. Mineral..

[41] Verweij H., Konijnendijk W. L. (1976). Structural units in K_2_O–PbO–SiO_2_ glasses by Raman spectroscopy. J. Am.
Ceram. Soc..

[42] Robinet L., Bouquillon A., Hartwig J. (2008). Correlations between Raman parameters
and elemental composition in lead and lead alkali silicate glasses. J. Raman Spectrosc..

[43] Pavić L., Narasimha Rao N., Moguš-Milanković A., Santic A., Ravi Kumar V., Piasecki M., Kityk I. V., Veeraiah N. (2014). Physical properties
of ZnF_2_–PbO–TeO_2_:TiO_2_ glass ceramics—Part III: Dielectric
dispersion and ac conduction phenomena. Ceram.
Int..

[44] Prasad V., Pavić L., Moguš-Milanković A., Siva Sesha Reddy A., Gandhi Y., Ravi Kumar V., Naga Raju G., Veeraiah N. (2019). Influence of silver ion concentration
on dielectric characteristics of Li_2_O–Nb_2_O_5_–P_2_O_5_ glasses. J. Alloys Compd..

[45] Reddy A. S. S., Brik M. G., Kumar J. S., Graça M. P. F., Raju G. N., Kumar V. R., Piasecki M., Veeraiah N. (2016). Structural
and electrical properties of zinc tantalum borate glass ceramic. Ceram. Int..

[46] Sambasiva
Rao K., Srinivasa Reddy M., Ravi Kumar V., Veeraiah N. (2007). Dielectric spectra of Li_2_O–CaF_2_–P_2_O_5_ glasses doped by silver
ions. Phys. B.

[47] Reddy M. R., Kumar V. R., Veeraiah N., Rao A. V. (1995). Effect
of chromium
impurity on dielectric relaxation effects of ZnF_2_–PbO–TeO_2_ glasses. Indian J. Pure Appl. Phys..

[48] Naresh P., Raju G. N., Kumar V. R., Piasecki M., Kiytyk I. V., Veeraiah N. (2014). Optical and dielectric
features of zinc oxyfluoro borate
glass ceramics with TiO_2_ as crystallizing agent. Ceram. Int..

[49] Long G. G., Cotton F. A. (1965). Stereochemically
active lone pairs. Inorg. Chem..

[50] Bottcher, C. J. F. Theory of Electrical Polarisation, Part II; Elsevier Publ. Co: NY, 1978.

[51] Srinivasa
Reddy M., Prasad S. V. G. V. A., Veeraiah N. (2007). Valence and coordination
of chromium ions in ZnO–Sb_2_O_3_–B_2_O_3_ glass system by means of spectroscopic and dielectric
relaxation studies. Phys. Status Solidi A.

[52] Elliott, S. R. Physics of Amorphous Materials; Longman: NY, 1985.

[53] Minakshi M., Aughterson R., Sharma P., Sunda A. P., Ariga K., Shrestha L. K. (2025). Micelle-Assisted Electrodeposition of γ-MnO_2_ on Lead Anodes: Structural and Electrochemical Insights. ChemNanoMat.

